# Non-Pharmacological Prehabilitation in Surgical Patients with Pre-Existing Chronic Cardio-Respiratory Disease: A Clinically Oriented Narrative Review

**DOI:** 10.3390/healthcare14152301

**Published:** 2026-07-30

**Authors:** Daniele Salvatore Paternò, Luigi La Via, Francesca Barbagallo, Flavia Arena, Paolo Tummino, Stefano Soriano, Emilia Concetta Lo Giudice, Sara Clelia Longo, Francesco Pegreffi, Sofia Miccichè, Fabrizio Luca, Giuseppe Scibilia, Massimiliano Sorbello

**Affiliations:** 1 Department of Anesthesia and Intensive Care, “Giovanni Paolo II” Hospital, 97100 Ragusa, Italy; stefano.soriano@asp.rg.it (S.S.); massimiliano.sorbello@unikore.it (M.S.); 2 Department of General Surgery and Medical-Surgical Specialties, University of Catania, 95123 Catania, Italy; luigi.lavia@unict.it; 3 School of Anesthesia and Intensive Care, “Kore” University, 94100 Enna, Italy; francesca.barbagallo002@unikorestudent.it (F.B.); flaviapaola.arena@unikorestudent.it (F.A.); paolo.tummino@unikorestudent.it (P.T.); emilia.logiudice@unikore.it (E.C.L.G.); 4 School of Medicine and Surgery, “Kore” University, 94100 Enna, Italy; francesco.pegreffi@unikore.it (F.P.); sofia.micciche028@unikorestudent.it (S.M.); giuseppe.scibilia@unikore.it (G.S.); 5 Recovery and Functional Rehabilitation Unit, “Giovanni Paolo II” Hospital, 97100 Ragusa, Italy; saraclelia.longo@asp.rg.it; 6 School of General Surgery, “Kore” University, 94100 Enna, Italy; 7 School of Obstetric and Gynecological Surgery, “Kore” University, 94100 Enna, Italy

**Keywords:** preoperative optimization, inspiratory muscle training, pulmonary rehabilitation, chronic obstructive pulmonary disease, heart failure, postoperative pulmonary complications, perioperative medicine

## Abstract

**Background/Objectives:** Patients with pre-existing chronic obstructive pulmonary disease (COPD) and/or chronic heart failure (CHF) are a particularly high-risk surgical population in whom postoperative pulmonary complications (PPCs) drive a disproportionate share of morbidity, prolonged stay and mortality. Prehabilitation—the structured optimization of functional reserve during the preoperative interval—has emerged as a proactive, largely non-pharmacological strategy to raise that reserve before the surgical insult. Yet the evidence base is organized predominantly by surgical procedure and enrolled in mixed populations, so the patients with the least physiological reserve—those with established COPD or CHF—remain comparatively understudied. This narrative review takes the comorbidity, rather than the incision, as its organizing axis. **Methods:** We summarize the pathophysiology that links chronic cardio-respiratory disease to perioperative respiratory failure; appraise the principal non-pharmacological interventions, with the respiratory components (inspiratory muscle training, pulmonary rehabilitation, breathing techniques) as the core and exercise, nutritional, psychological, and smoking-cessation elements as the multimodal context; and re-read the surgical evidence through the lens of the underlying disease. **Results:** A cross-surgical meta-analysis indicates that preoperative exercise training reduces PPCs (relative risk ≈ 0.52) with no significant difference in effect across surgery type or training modality, supporting a comorbidity-centered rather than procedure-centered framework. We address practical determinants of implementation—timing, patient selection, adherence, home-based and telemonitored delivery, and low-resource applicability—and highlight two cross-cutting problems: heterogeneity of intervention prescription and the lack of standardized PPC definitions. **Conclusions:** The scarcity of disease-specific evidence is itself the central finding, revealing a mismatch between clinical risk and research investment.

## 1. Introduction

Surgical volume in ageing populations increasingly includes patients with chronic cardio-respiratory disease. For the purposes of this review, we define that population as adults with established COPD and/or CHF—two conditions that share risk factors and frequently coexist, with heart failure found in 10–46% of COPD patients and COPD in roughly 20% of heart failure patients, and are each independent predictors of perioperative morbidity and mortality [1,2,3]. Among postoperative adverse events, PPCs are common and carry a heavy burden in length of stay, intensive care utilization and death; their reported incidence ranges from a few percent to roughly a third of patients, varying chiefly with the definition applied and the population studied [4,5,6].

**Figure 1:**
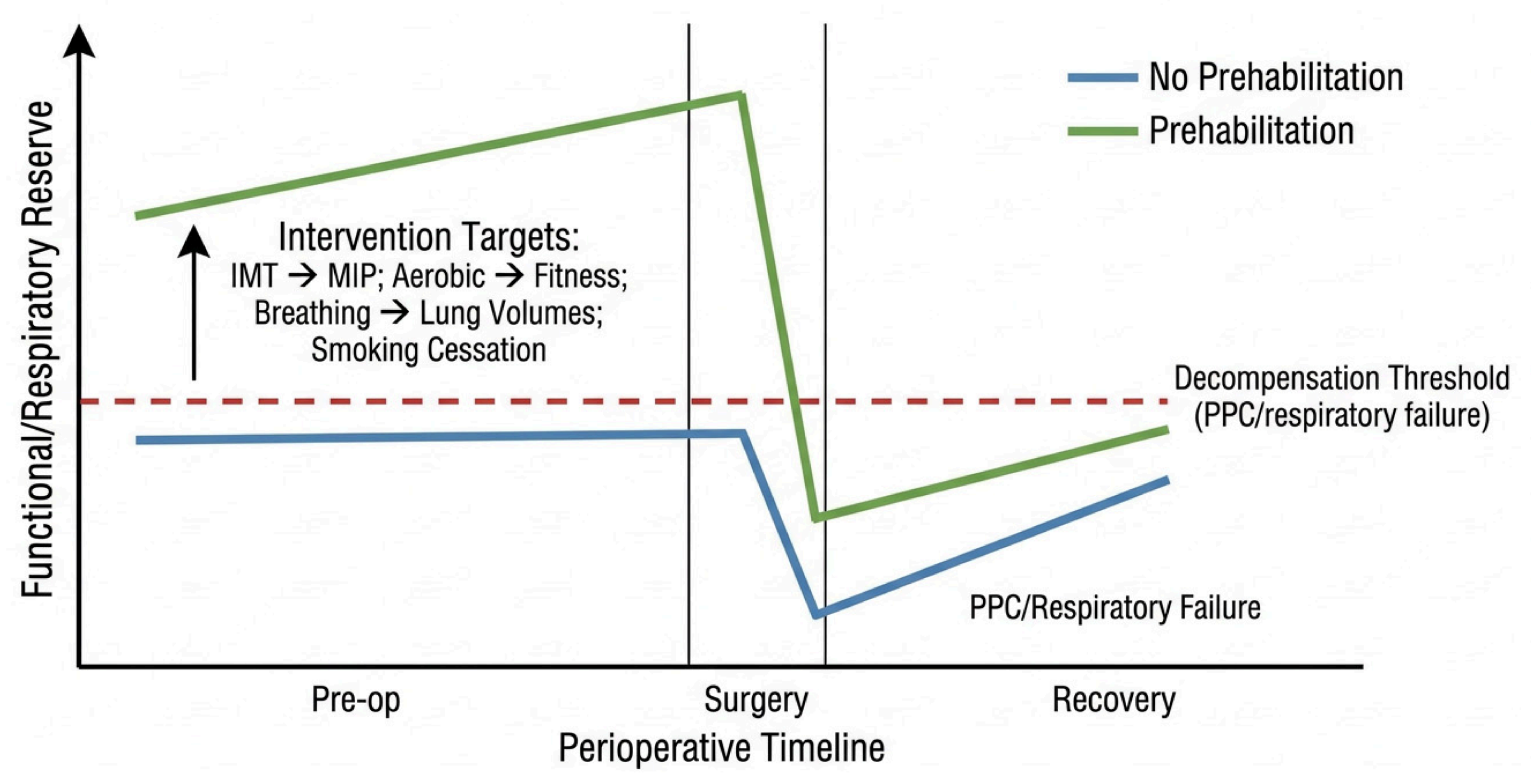
Functional reserve and the decompensation threshold in the chronic cardio-respiratory surgical patient. Prehabilitation does not change the magnitude of the surgical insult; it raises the functional floor from which the patient absorbs it, so that the postoperative nadir remains above the threshold for postoperative pulmonary complications and respiratory failure. Intervention targets during the preoperative window: inspiratory muscle training (IMT) → maximal inspiratory pressure (MIP); aerobic training → fitness; breathing techniques → lung volumes; smoking cessation. IMT, inspiratory muscle training; MIP, maximal inspiratory pressure; PPC, postoperative pulmonary complication.

The preoperative period is a window of opportunity. Unlike rehabilitation, delivered after the physiological insult, prehabilitation aims to raise the patient’s functional baseline beforehand, so that the inevitable postoperative decline begins from a higher point and crosses the threshold of clinical decompensation less often (Figure 1) [7]. In the chronic cardio-respiratory patient, the targets are concrete: the inspiratory muscle pump, ventilatory efficiency, exercise capacity and airway clearance—the functions most compromised after thoracic, upper-abdominal and cardiac procedures.

A substantial literature supports preoperative exercise and respiratory training, but it is structured chiefly around the type of operation—lung resection, cardiac surgery, major abdominal surgery—and enrolled in mixed surgical samples in which the subgroup with established COPD or CHF is diluted. This represents an important clinical paradox: the patients with the least reserve, in whom prehabilitation should plausibly yield the greatest absolute benefit, are precisely those for whom dedicated evidence is sparsest. We do not claim that diminished physiological reserve is unique to this group—prehabilitation is also of proven value in other low-reserve populations, notably patients with cancer; rather, our argument is that among common surgical comorbidities, COPD and CHF combine high prevalence, frequent coexistence, a shared final common pathway of impaired ventilatory and cardiac reserve, and a disproportionate PPC burden, yet attract the least disease-specific prehabilitation evidence. We therefore group COPD and CHF not because their pathophysiology is identical—it is not—but because they converge on the same perioperative vulnerability and are addressed through an overlapping non-pharmacological toolkit; where the evidence base and the optimal prescription diverge between the two conditions, we make that distinction explicit. A feasibility study of preoperative pulmonary rehabilitation in patients with COPD scheduled for major abdominal surgery makes the point starkly—the investigators concluded that a definitive randomized trial was not feasible because eligible patients could not be reliably identified and recruited within the surgical pathway [8]. A related population—waitlisted candidates for solid-organ (including heart and lung) transplantation—also undergoes structured prehabilitation, and a recent European Society for Organ Transplantation consensus statement offers recommendations in that setting [9]; although these patients fall outside the elective-surgical scope of this review, the underlying rationale, raising functional reserve before an unavoidable physiological insult, is shared.

This review therefore takes the comorbidity, not the incision, as its organizing axis. We (i) outline the pathophysiology that makes the chronic cardio-respiratory patient vulnerable to perioperative respiratory failure; (ii) describe the principal non-pharmacological prehabilitation interventions and their mechanisms, foregrounding the respiratory components; (iii) re-read the surgical evidence by underlying disease; (iv) discuss the practical determinants of implementation; and (v) identify the gaps that a focused research agenda should address.

### Approach to the Literature

This is a narrative, clinically oriented review and does not constitute a systematic review; we did not pre-register a protocol or perform duplicate screening. To limit selection bias, we followed the principles of the Scale for the Assessment of Narrative Review Articles (SANRA). We searched PubMed/MEDLINE (inception to May 2026) for English- and Italian-language randomized trials, systematic reviews, meta-analyses and consensus statements on preoperative inspiratory muscle training, pulmonary rehabilitation, breathing exercises, aerobic and multimodal prehabilitation, smoking cessation, and on the definition and prediction of PPCs, prioritizing the highest-level and most recent evidence and, wherever available, studies enrolling patients with COPD or CHF. Selection was purposive and was guided by the pre-specified eligibility criteria set out below and by the highest available level of evidence; we recognize that this expert-guided, non-exhaustive approach carries a risk of selection and citation bias, which we list among the study’s limitations (Section Limitations). Studies were considered eligible when they addressed non-pharmacological preoperative optimization in adults undergoing surgery, with priority given to those involving chronic cardio-respiratory disease; mechanistic, pathophysiological and guideline sources were additionally included to provide clinical context. The principal search combined terms for the interventions (“prehabilitation” OR “preoperative exercise” OR “inspiratory muscle training” OR “pulmonary rehabilitation” OR “breathing exercises” OR “multimodal prehabilitation” OR “smoking cessation”) with terms for the setting (“surgery” OR “perioperative” OR “postoperative pulmonary complications”) and, where relevant, for the comorbidity (“COPD” OR “chronic obstructive pulmonary disease” OR “heart failure”), supplemented by backward citation-tracking of the reference lists of the retrieved reviews. Studies were eligible if they were randomized controlled trials, systematic reviews, meta-analyses or consensus statements addressing non-pharmacological preoperative optimization in adults undergoing surgery; we excluded conference abstracts, opinion pieces without primary data, reports not available in English or Italian, and studies confined to purely postoperative rehabilitation. We deliberately restricted the primary search to PubMed/MEDLINE because its indexing of the clinical perioperative and rehabilitation literature is comprehensive for the high-level designs we prioritized and because a single-database, purposive strategy is appropriate to a clinically oriented narrative synthesis rather than a systematic review; reliance on a single database is nonetheless acknowledged as a limitation (Section Limitations). Consistent with this narrative design, we did not generate formal record counts or a study-selection flow diagram, which would imply a systematic methodology that was not undertaken.

## 2. Pathophysiology of Perioperative Respiratory Risk in Chronic Cardio-Respiratory Disease

Perioperative respiratory vulnerability in these patients arises from the collision of three processes: a reduced baseline reserve, the respiratory consequences of anesthesia and surgery, and an impaired capacity to recover (Figure 1).

Reduced baseline reserve. In COPD, expiratory flow limitation, dynamic hyperinflation and gas-exchange abnormality coexist with skeletal and respiratory muscle dysfunction and systemic deconditioning [1]; chronic hyperinflation flattens the diaphragm and shifts it onto an unfavorable portion of its length–tension relationship, mechanically disadvantaging the principal inspiratory muscle [10]. In CHF, low cardiac output, pulmonary congestion and ventilatory inefficiency limit the response to increased ventilatory demand [2]. Both conditions converge on weakness of the inspiratory muscles—and this convergence is the mechanistic rationale for respiratory prehabilitation. In CHF specifically, maximal inspiratory pressure (Pimax/MIP) is reduced relative to controls and is an independent predictor of mortality, with worse survival across descending Pimax quartiles [11]. Because MIP is modifiable—it rises with targeted training even in CHF [12]—it is both a therapeutic target and a marker of the capacity to cough, clear secretions and sustain spontaneous ventilation after extubation.

The surgical and anesthetic insult. General anesthesia, neuromuscular blockade, supine positioning and incisions near the diaphragm reduce functional residual capacity, promote atelectasis—seen in over three-quarters of patients receiving a neuromuscular blocking drug—and impair mucociliary clearance, with the respiratory system potentially requiring several weeks to return toward baseline function after major surgery [6]. For PPCs, surgical-site and procedural factors—upper-abdominal and intrathoracic incisions, prolonged operative time, nasogastric intubation—are dominant acute determinants of risk [13]. Crucially, this does not diminish the role of comorbidity: the chronic disease sets the floor of reserve from which the patient must absorb that procedural hit. The chronic cardio-respiratory patient therefore faces a double jeopardy—a high procedural load superimposed on a low baseline—which is precisely why raising the floor beforehand is attractive.

Impaired recovery. Postoperative pain, diaphragmatic dysfunction and immobility further depress lung volumes and clearance, while in CHF perioperative fluid shifts threaten congestion. In this setting, a modest preoperative functional gain may translate into a clinically meaningful reduction in the probability of crossing into respiratory failure.

These mechanisms define the rationale for prehabilitation: strengthen the inspiratory pump (IMT), improve ventilatory efficiency and exercise capacity (aerobic and pulmonary rehabilitation), optimize secretion clearance and lung expansion (breathing techniques), and remove reversible aggravators (tobacco exposure, deconditioning, malnutrition).

## 3. Non-Pharmacological Prehabilitation Interventions

The respiratory components are the core of this review; exercise, multimodal and smoking-cessation elements are included because they form the realistic clinical package within which respiratory prehabilitation is delivered. The interventions below are summarized in Table 1.

### 3.1. Inspiratory Muscle Training (IMT)

IMT applies a controlled inspiratory load—usually via a threshold or tapered flow-resistive device—to strengthen and increase the endurance of the inspiratory muscles. The landmark evidence comes from cardiac surgery: in a randomized trial of high-risk patients scheduled for elective coronary artery bypass grafting (CABG), preoperative IMT reduced PPCs versus usual care [14]; a preceding pilot had shown that 2–4 weeks of IMT was well tolerated and associated with fewer atelectases [15]. Subsequent synthesis broadly supports these findings while exposing their limits. A Cochrane review of preoperative IMT before cardiac and major abdominal surgery concluded that training may reduce PPCs and shorten stay, with the caveat of heterogeneous protocols and variable quality [16]. A meta-analysis with trial sequential analysis (13 trials, 784 patients) reported a reduction in PPCs (relative risk 0.59, 95% CI 0.47–0.74) and a gain in MIP, with a signal of dose-dependence on session duration [17]; a focused systematic review concurred for cardiac surgery [18]. A recurring debate concerns training intensity (high- versus low-intensity loading), but the optimal prescription remains undefined. The CHF evidence is instructive here: a landmark randomized trial of 12-week IMT in patients with CHF and inspiratory muscle weakness improved MIP, peak oxygen uptake and quality of life [12], and meta-analyses confirm gains in inspiratory strength, exercise capacity and quality of life—largest where baseline weakness is present, which argues for targeting and stratifying by MIP [19,20]. Indeed, the prescription itself—load (% MIP), session volume, frequency, total duration, progression rule and supervision—is markedly non-standardized, an issue we return to in Section 6.

### 3.2. Pulmonary Rehabilitation and Aerobic Exercise Training

Pulmonary rehabilitation combines aerobic and resistance exercise with education and, often, breathing retraining. Transferred to the preoperative setting, exercise-based prehabilitation aims to raise cardio-respiratory fitness, a strong correlate of postoperative outcome. In operable lung cancer—a population heavily enriched for COPD—a meta-analysis of randomized trials found that preoperative exercise reduced postoperative complications and shortened stay while improving pulmonary function and exercise capacity [21], and a Cochrane review reported favorable effects on exercise capacity and PPCs, tempered by study-quality concerns [22,23]. When the therapeutic quality of programs was formally appraised, prehabilitation still reduced PPCs (odds ratio ≈ 0.45) and severe complications, but half the programs were judged at high risk of being ineffective owing to inadequate dose reporting, poor adherence or mismatched outcome assessment [24]. Short, intensive programs of one to two weeks have improved outcomes even in patients with limited lung function, indicating feasibility when time is short [25,26].

### 3.3. Breathing Techniques and Lung-Expansion Maneuvers

Breathing exercises, directed coughing and incentive spirometry aim to preserve or restore lung volumes and clearance. The evidence is mixed and method-dependent, but these techniques are inexpensive and low-risk, which makes them attractive components of multimodal programs and of low-resource implementations even where high-certainty efficacy data are lacking [27].

### 3.4. Multimodal Prehabilitation: The Delivery Context

Multimodal prehabilitation integrates exercise with nutritional optimization and psychological support, typically within Enhanced Recovery After Surgery (ERAS) pathways. In colorectal surgery, meta-analytic data indicate reduced overall complications (odds ratio ≈ 0.60) and improved six-minute walk distance, though effects on severe complications are less certain [28]; a multicenter randomized trial reported reduced complications with multimodal prehabilitation [29], while other trials—including in frail elderly cohorts—found no reduction in 30-day complications, reflecting heterogeneous populations, protocols and adherence [30,31]. Evidence on esophagogastric cancer suggests that programs with a mandatory nutritional component reduce complications, most consistently when multimodal rather than single-modality [32]. For the COPD or CHF patient, the respiratory components are the logical anchor of such a program—and in CHF, adding IMT to aerobic training yields additive cardio-respiratory gains [33]—but dedicated trials in this comorbid subgroup remain scarce.

### 3.5. Smoking Cessation as an Adjunctive Measure

Many patients with COPD are current or recent smokers, and the preoperative encounter is a teachable moment. A Cochrane review found that preoperative smoking-cessation interventions increase short-term abstinence and reduce postoperative complications, with intensive behavioral support more effective than brief advice [34]. Although not a respiratory-training technique, smoking cessation is a high-yield, non-pharmacological lever that belongs in any prehabilitation pathway for this population, and is strongly endorsed in current ERAS guidance [35].

### 3.6. Rehabilitation Assessment and Individualized Prescription

From a rehabilitation perspective, prehabilitation should not be conceived as a generic exercise package, but as a time-limited, impairment-oriented intervention prescribed according to baseline reserve, dominant functional limitation and surgical urgency [7,36]. In patients with COPD or CHF, a structured rehabilitation assessment should precede the intervention whenever feasible. This assessment may include dyspnea severity, exercise tolerance, inspiratory muscle strength, peripheral muscle function, nutritional risk, frailty, activities of daily living and barriers to adherence. Practical tools such as the six-minute walk test, the one-minute sit-to-stand test, the modified Medical Research Council dyspnea scale, maximal inspiratory pressure measurement and peripheral oxygen saturation monitoring during exertion can help tailor the intervention to the individual patient, complementing formal perioperative risk stratification [5].

In COPD, the rehabilitation focus should include inspiratory muscle loading, aerobic reconditioning, lower-limb strengthening, breathing control, airway-clearance strategies when secretions are present, and education on exacerbation recognition and perioperative self-management [1]. In CHF, prehabilitation should be restricted to clinically stable patients and should emphasize symptom-limited aerobic training, low-to-moderate resistance exercise, inspiratory muscle training—particularly when inspiratory weakness is documented [11,12,19]—and close monitoring of dyspnea, fatigue, weight change, congestion symptoms and exercise tolerance [2].

The rehabilitation specialist may therefore contribute to perioperative prehabilitation by translating risk stratification into an individualized functional prescription; identifying modifiable impairments such as inspiratory muscle weakness, deconditioning, impaired airway clearance and frailty; selecting feasible supervised, home-based or telemonitored strategies [37,38]; monitoring adherence and safety; and adapting the program when the surgical window is short. Throughout these recommendations, we distinguish statements grounded in randomized or meta-analytic evidence—principally the efficacy of IMT and exercise-based prehabilitation for reducing PPCs in mixed and cardiac/thoracic populations—from those that rest on extrapolation from adjacent populations or on expert opinion, such as the specific composition of an individualized prescription in isolated COPD or CHF, which remains largely consensus-based.

**Table 1:** Non-pharmacological prehabilitation interventions in the chronic cardio-respiratory surgical patient.

Intervention	Core Mechanism	Representative Evidence (Effect)	Strongest Surgical Context	COPD/CHF-Specific Data	Certainty of Evidence †
Inspiratory muscle training (IMT)	↑ inspiratory muscle strength/endurance (MIP); better cough and post-extubation ventilation	RR for PPCs ≈ 0.59 (meta-analysis, TSA) [17]; landmark CABG RCT [14]	Cardiac, upper abdominal	Physiological target well-defined in CHF (low MIP, prognostic) [11]; few dedicated trials	Moderate (multiple RCTs and meta-analyses; downgraded for protocol heterogeneity)
Pulmonary rehabilitation/aerobic training	↑ cardio-respiratory fitness, exercise capacity	↓ PPCs and LOS in lung cancer [21,22,23]; OR ≈ 0.45 when quality-appraised [24]	Thoracic/lung resection (COPD-enriched)	Indirect (lung-cancer cohorts with COPD); sparse in isolated COPD/CHF	Low–moderate (RCTs and Cochrane reviews; downgraded for study quality and indirectness)
Breathing techniques/incentive spirometry	preserve/restore lung volumes and clearance	mixed, method-dependent; low-risk, low-cost [27]	Abdominal, thoracic	limited	Low (small, method-dependent trials; imprecision)
Multimodal (exercise + nutrition + psychology)	composite physiological + nutritional reserve	↓ overall complications OR ≈ 0.60 (colorectal) [28,29]; conflicting in frail cohorts [30,31]	Colorectal, esophagogastric	scarce in comorbid subgroup	Low–moderate (RCTs and meta-analyses; inconsistency across cohorts)
Smoking cessation	removes reversible airway/wound risk factor	↑ abstinence, ↓ complications [34]; strong ERAS recommendation [35]	all	directly relevant to COPD	Moderate (Cochrane review of RCTs; well established)
Cross-surgical synthesis	—	RR for PPCs ≈ 0.52; no difference in effect across surgery type, modality or duration [39]	all major surgery	supports comorbidity- over procedure-based framing	Moderate (large meta-analysis with TSA; indirect for isolated COPD/CHF)

CABG, coronary artery bypass grafting; CHF, chronic heart failure; COPD, chronic obstructive pulmonary disease; LOS, length of stay; MIP, maximal inspiratory pressure; OR, odds ratio; PPC, postoperative pulmonary complication; RR, relative risk; TSA, trial sequential analysis. † Certainty reflects a qualitative appraisal of study design, consistency and directness, not a formal GRADE assessment. ↑, increase; ↓, decrease.

## 4. Evidence by Comorbidity and Across Surgical Settings

A central argument of this review is that the underlying disease, not the operation, is the more informative axis—and the data support reading the evidence this way. A cross-surgical meta-analysis with trial sequential analysis (29 trials, 2070 patients) found that preoperative exercise training reduced PPCs (relative risk 0.52, 95% CI 0.41–0.66) with no significant difference in effect across surgery type, training modality, or training duration, alongside gains in peak oxygen uptake and inspiratory pressure [39]. If the effect is consistent across procedures, then organizing the evidence by incision obscures more than it reveals; the patient’s reserve is the variable that travels across settings.

### 4.1. The Patient with COPD

COPD is an independent risk factor for postoperative pulmonary and cardiac complications and is over-represented among surgical—particularly cardiac surgical—populations relative to the general population [13,40], a setting in which recent meta-analytic evidence supports prehabilitation before cardiac procedures [41]; a propensity-score-matched cohort likewise confirms worse postoperative outcomes—including higher mortality and prolonged intensive care and hospital stay—in COPD patients undergoing elective non-cardiac surgery [42]. The intervention evidence most applicable to these patients comes from lung resection (where COPD prevalence is high): preoperative exercise and pulmonary rehabilitation improve fitness and reduce PPCs and length of stay, including in patients with limited lung function [21,22,23,43], and short high-intensity programs are pragmatic when oncological timelines compress the window [25,26]. In major abdominal surgery, IMT and physiotherapy improve pulmonary function and reduce complications [16,17,27,44], yet the COPD-specific feasibility data are sobering: identifying and recruiting patients with established COPD early enough in the abdominal pathway proved impractical in a dedicated study—a barrier as much organizational as physiological [8].

### 4.2. The Patient with CHF

Patients with CHF represent the least studied subgroup. Inspiratory muscle weakness is prevalent in CHF and carries independent prognostic weight [11], providing a clear physiological target; in stable CHF, IMT improves inspiratory strength, peak oxygen uptake and quality of life outside the surgical setting, with the greatest benefit in patients who start with demonstrable weakness [12,19,20]. In the perioperative context, however, most evidence derives from cardiac surgical cohorts rather than from CHF patients undergoing non-cardiac surgery, leaving a conspicuous gap. A 2024 scoping review of respiratory muscle training before and after cardiac surgery identified only a small number of eligible studies, underscoring how thin the high-quality, procedure-specific evidence remains even in the most-studied surgical setting [45]. For the CHF patient facing non-cardiac major surgery—arguably the population in greatest need, and one in which heart failure independently predicts higher perioperative morbidity and mortality [46]—dedicated prehabilitation evidence is effectively absent, and exercise prescription must in any case respect clinical stability and standard cardiac-rehabilitation safeguards. Prioritization of the evidence in this section reflects the paucity of dedicated data: we drew first on randomized trials and meta-analyses of IMT and exercise in stable CHF outside surgery [12,19,20,33], then on perioperative cardiac-surgical cohorts, and finally on indirect inference for the CHF patient facing non-cardiac surgery, for whom direct trials are lacking. Cardiac rehabilitation—an established, guideline-endorsed program of supervised aerobic and resistance training, risk-factor modification and education in stable heart failure—provides the natural delivery framework for CHF prehabilitation, and its safeguards (symptom-limited intensity, monitoring of volume status and arrhythmia) should govern any preoperative exercise in these patients. Because the interventions, monitoring and contraindications differ materially between COPD and CHF, we present disease-specific prescriptions separately (Section 3.6 and Table 1) rather than treating the two conditions interchangeably.

Across both diseases, a consistent theme emerges: the interventions work in principle and in mixed populations, the cross-surgical effect is homogeneous, but evidence selected specifically for pre-existing COPD or CHF is limited and is further weakened by heterogeneous prescriptions and inconsistent outcome definitions. It is equally important not to over-read the positive literature: several well-conducted trials report no reduction in complications—for example, multimodal prehabilitation in frail elderly and mixed colorectal cohorts [30,31]—and the 2025 ERAS colorectal update withdrew its prehabilitation recommendation on the grounds of inconsistent and heterogeneous evidence [47]. These neutral and cautionary signals, together with the likelihood of publication bias favoring positive studies, temper the strength of any recommendation for the comorbid population.

## 5. Practical Considerations for Implementation

Timing and duration. The preoperative window in these patients is often short and contested by oncological or symptomatic urgency. Benefit has been shown across programs from a single week to several weeks [17,25,26,39]; the practical task is to match an achievable dose to the available interval rather than default to a fixed multi-week protocol that surgery will not wait for. Where time is minimal, an intensive short course of IMT (for example, daily threshold loading at a defined percentage of MIP with weekly re-titration) or of exercise may be the only realistic option.

Patient selection and pathway integration. The feasibility literature shows that the rate-limiting step is frequently not the training but the timely identification of eligible high-risk patients [8]. Embedding risk stratification at the point of surgical listing—using a validated tool such as ARISCAT and explicitly flagging COPD/CHF—may be a prerequisite for effective prehabilitation rather than an optional refinement [5].

Adherence and delivery models. Supervised hospital-based programs maximize fidelity but compete with travel burden and service capacity. Home-based and telemonitored delivery can extend reach and is particularly relevant for comorbid patients with mobility limitation: a meta-analysis of 29 randomized trials of home-based prehabilitation reported good median adherence (≈82%) and a reduction in postoperative complications, provided adherence is measured and reported—something current studies still do inconsistently [37]. A multicenter tele-prehabilitation randomized trial in elective cardiac surgery, delivering a multimodal online program that included inspiratory muscle training, reduced major adverse cardiovascular events and lowered the preoperative prevalence of active smoking and elevated pulmonary-risk scores, demonstrating that meaningful optimization can be achieved remotely [38]. Emerging digital approaches are likely to reshape delivery: wearable activity and physiological sensors allow objective monitoring of adherence and training load; smartphone platforms and tele-prehabilitation extend supervised programs beyond the hospital [37,38]; and machine-learning models applied to routine preoperative data are being explored for automated risk stratification that could direct prehabilitation to those most likely to benefit. Evidence for these tools in the comorbid population remains preliminary, and they should be regarded as promising adjuncts rather than established standards.

Low-resource settings. Several core components—threshold IMT devices, breathing techniques, walking-based aerobic training, structured smoking cessation—are inexpensive and scalable, making prehabilitation plausible even where formal rehabilitation infrastructure is limited, and home-based models specifically have been shown to be feasible and effective without consuming hospital resources [37]. This aligns with the broader agenda of equitable, non-pharmacological care for chronic cardio-respiratory disease that motivates this Special Issue. Because COPD and CHF are globally prevalent and the surgical burden of these comorbidities is rising fastest in low- and middle-income settings, a scalable, low-cost, largely non-pharmacological prehabilitation package has particular global-health relevance, offering benefit that is not contingent on high-technology perioperative infrastructure.

Safety. Across the trial literature, prehabilitation is generally well tolerated, and serious adverse events are uncommon [16,17,39]. In CHF, exercise must be prescribed only in clinically stable patients, with attention to volume status, arrhythmia and symptom-limited intensity, and ideally within a cardiac-rehabilitation framework; in severe COPD, exacerbation during the training window should trigger reassessment and, where appropriate, deferral.

## 6. Gaps and Future Directions

Two methodological problems cut across the field and disproportionately affect the comorbid population.

Prescription heterogeneity. The “active ingredient” of prehabilitation—load, volume, frequency, progression, supervision and total dose—is reported inconsistently and varies widely, limiting comparability and reproducibility and weakening meta-analytic precision [16,17,24]. The clinical stakes are not abstract: in its most recent (2025) update, the ERAS Society colorectal guideline withdrew its recommendation for prehabilitation, judging that it can no longer be recommended on current evidence given inconsistent definitions and heterogeneous trials [47]—a caution echoed by an umbrella review of the wider prehabilitation literature [36], and one that applies with particular force to the under-studied comorbid population considered here. A minimum reporting dataset for prehabilitation prescription (device, % MIP or training intensity, sets/repetitions, frequency, duration, progression rule, supervision and measured adherence), building on existing intervention- and exercise-reporting frameworks such as TIDieR and CERT [48,49], would be a high-value, low-cost contribution.

Outcome definition heterogeneity. PPCs have been defined in numerous, often poorly specified ways. The StEP-COMPAC consensus proposed standardized, severity-graded definitions precisely to address this [4], and risk-prediction work confirms that estimated PPC rates shift substantially with the definition used [5]. Future prehabilitation trials should adopt standardized PPC endpoints to allow pooling. Finally, this field—and the present review—has focused almost exclusively on PPCs; future work should broaden the outcome set to capture quality of recovery, functional recovery, patient-reported outcomes, hospital readmission and healthcare utilization, dimensions of benefit that are especially relevant to comorbid patients yet remain rarely reported.

The comorbidity-specific evidence gap. Most importantly, the population at greatest physiological risk—patients with established COPD and/or CHF, especially CHF patients undergoing non-cardiac surgery—is the least represented in dedicated trials, and the feasibility barriers to studying them are real [8]. The scarcity of focused evidence is itself a finding: it reveals a mismatch between clinical risk and research investment. Priorities include (i) pragmatic, pathway-embedded trials enrolling diagnosed COPD/CHF patients across surgery types, ideally stratified by baseline MIP and fitness; (ii) standardized, severity-graded PPC outcomes; (iii) a shared minimum prescription-reporting dataset; (iv) evaluation of home-based and telemonitored delivery with explicit adherence metrics; and (v) a multicenter prehabilitation registry for this comorbid population to accumulate data where randomized recruitment is not feasible. Concretely, we propose a multicenter, pathway-embedded randomized (or registry-based pragmatic) trial enrolling adults with spirometrically confirmed COPD and/or echocardiographically defined CHF across surgery types, stratified by baseline MIP and functional capacity, comparing a standardized minimum prehabilitation package against usual care; a core outcome set built on StEP-COMPAC severity-graded PPCs [4] together with days-alive-and-at-home, quality of recovery and patient-reported function; and adherence to TIDieR and CERT reporting standards [48,49] as a condition of publication.

### Limitations

This review has several limitations. It is a narrative, clinically oriented synthesis rather than a systematic review: we did not register a protocol, perform duplicate screening, or conduct a database-exhaustive search, and the primary search was restricted to PubMed/MEDLINE and to English- and Italian-language records, so relevant studies indexed elsewhere or reported in other languages may have been missed. Study selection was expert-guided and purposive, which—despite the pre-specified eligibility criteria—carries a risk of selection and citation bias. We did not undertake a formal risk-of-bias or GRADE certainty assessment; the certainty-of-evidence and key-limitation entries in Table 1 and Table 2 therefore provide a structured qualitative appraisal rather than a quantitative one. Finally, much of the evidence we apply to the COPD and CHF patient is extrapolated from mixed surgical populations rather than derived from disease-specific trials, and this indirectness—rather than any single numerical estimate—should frame how the recommendations are read.

## 7. Conclusions

Patients with pre-existing chronic cardio-respiratory disease are simultaneously the most vulnerable to postoperative respiratory failure and, in principle, the most likely to benefit from preoperative optimization—yet they remain under-represented in the dedicated evidence base, the CHF patient undergoing non-cardiac surgery most of all. Non-pharmacological prehabilitation—anchored by inspiratory muscle training, pulmonary rehabilitation and breathing techniques, and delivered within a multimodal package—is supported by trial and meta-analytic evidence whose effect appears consistent across surgical settings, and it is generally safe, inexpensive and scalable. The path from this general promise to the individual comorbid patient runs through three practical commitments: identify these patients early and stratify them by reserve; standardize both what is prescribed and what is measured; and build the comorbidity-specific evidence—through pragmatic trials and registries—that the most at-risk surgical patients currently lack. Until then, the strongest single message for clinical teams is also the simplest: the patient with the least reserve is the one in whom raising the floor before surgery is most worth attempting.

## Figures and Tables

**Table 2:** Summary of the principal evidence for non-pharmacological prehabilitation: study design, population, intervention, outcomes, effect and certainty.

Study [Ref]	Design; Population (*n*)	Intervention; Duration	Main Outcome(s)	Key Finding (Effect)	Certainty; Key Limitation
Hulzebos 2006 [14]	RCT; high-risk pre-CABG cardiac surgery (*n* ≈ 279)	Preoperative IMT; ≥2 weeks	PPCs; LOS	Fewer PPCs and shorter stay vs. usual care	Moderate; single-center, unblinded
Katsura 2015 (Cochrane) [16]	Systematic review/meta-analysis; cardiac and major abdominal	Preoperative IMT	PPCs; LOS	May reduce PPCs and LOS	Low–moderate; heterogeneous protocols, variable quality
Ge 2018 [17]	Meta-analysis with TSA; 13 RCTs (*n* = 784)	Preoperative IMT	PPCs; MIP	RR 0.59 (0.47–0.74); dose–response on session duration	Moderate; heterogeneity in intensity
Sebio García 2016 [21]	Meta-analysis of RCTs; operable lung cancer (COPD-enriched)	Preoperative exercise/pulmonary rehabilitation	PPCs; LOS; fitness	Fewer PPCs, shorter LOS, improved capacity	Low–moderate; indirect for isolated COPD
Granger 2022 (Cochrane) [22]	Systematic review; NSCLC	Preoperative exercise	Exercise capacity; PPCs	Favorable effects, quality-limited	Low; study-quality concerns
Assouline 2021 [39]	Meta-analysis with TSA; 29 RCTs (*n* = 2070)	Preoperative exercise training	PPCs; VO2peak; MIP	RR 0.52 (0.41–0.66); no effect-modification by surgery type or modality	Moderate; mixed populations, indirect for COPD/CHF
Colorectal multimodal MA [28]	Meta-analysis; colorectal surgery	Multimodal prehabilitation (exercise + nutrition + psychology)	Overall complications; 6MWD	OR ≈ 0.60; less certain for severe complications	Low–moderate; heterogeneity, adherence
Frail/elderly and colorectal RCTs [30,31]	RCTs; frail elderly and mixed colorectal	Multimodal prehabilitation	30-day complications	No significant reduction (neutral trials)	Moderate; population heterogeneity
Dall’Ago 2006/Smart 2013 [12,19]	RCT and meta-analysis; stable CHF with inspiratory weakness (non-surgical)	IMT; ≈12 weeks	MIP; VO2peak; QoL	Improved, greatest with baseline weakness	Moderate; non-perioperative, indirect
Thomsen 2014 (Cochrane) [34]	Systematic review of RCTs	Preoperative smoking cessation	Abstinence; complications	↑ short-term abstinence; ↓ complications	Moderate; intensity-dependent

6MWD, six-minute walk distance; CABG, coronary artery bypass grafting; CHF, chronic heart failure; COPD, chronic obstructive pulmonary disease; IMT, inspiratory muscle training; LOS, length of stay; MA, meta-analysis; MIP, maximal inspiratory pressure; NSCLC, non-small-cell lung cancer; OR, odds ratio; PPC, postoperative pulmonary complication; QoL, quality of life; RCT, randomized controlled trial; RR, relative risk; TSA, trial sequential analysis; VO2peak, peak oxygen uptake. Certainty reflects a qualitative appraisal of study design, consistency and directness, not a formal GRADE assessment. ↑, increase; ↓, decrease.

## Data Availability

No new data were created or analyzed in this study. Data sharing is not applicable to this article.
